# A new method to confirm the absence of human and animal serum in mesenchymal stem cell culture media

**DOI:** 10.7150/ijms.32100

**Published:** 2019-08-06

**Authors:** Megumi Ota, Kentaro Takagaki, Sho Takaoka, Hideki Tanemura, Naoki Urushihata

**Affiliations:** BioMimetics Sympathies Inc., Aomi, Koto-Ku, Tokyo, Japan

**Keywords:** Human adipose-derived mesenchymal stem cells, BDKRB1 gene, serum-free culture medium, stable cell culture

## Abstract

Mesenchymal stem cells are an ideal source for regenerative medicine. For clinical use, cell culture should be done at stable conditions, thus the use of serum should be avoided because of the batch-to-batch variations of serum. Although several kinds of serum-free media are available, a method to confirm whether they contain serum has not been established yet. During studies on effect of adipocyte mesenchymal stem cells (Ad-MSCs) on pain using a human pain gene array, we noticed that BDKRB1 gene was constantly upregulated when serum was used in the culture medium. In this study, we attempted to establish further the potential of this gene as a new marker indicative of the presence of serum in media. Using a real-time quantitative PCR gene array screening containing 84 functional genes, we verified BDKRB1 as a specific gene upregulated in the presence of serum. The expression of BDKRB1 in Ad-MSCs was induced not only by bovine serum but also by human serum. The BDKRB1 expression was induced even when Ad-MSCs was cultured with 0.1% serum in the medium. We concluded that BDKRB1 is a valuable marker to detect traces of both human and animal serum in Ad-MSCs cultures. Our study provides a new method to confirm the absence of serum in media and ensure a stable cell culture condition.

## Introduction

Mesenchymal stem cells (MSCs) were discovered as adhesive multipotent cells in bone marrow 1. These cells can differentiate into mesenchymal tissues such as fat, bone, and cartilage 2. Cells with similar properties can be separated from other tissues such as fat and umbilical cord 3, 4. Unlike embryonic stem cells/induced pluripotent stem cells **(**ES/iPS), MSCs have extremely limited risk of cancer transformation 5. Among MSCs, adipose-derived MSCs (Ad-MSCs) can be isolated easily 3.

Nowadays, MSCs has attracted significant attention among researchers due to their potential clinical applications. MSCs have exhibited proven efficacy in some disorders, such as autoimmune disease and inflammation treatment 6, 7. However, there are some limitations with the use of MSCs. Conventionally, media containing supplementation of fetal bovine serum (FBS) at 10 to 20% (v/v) is used for isolating and expanding MSCs. Here, one major problem is the batch-to-batch variation that can affect cell growth or characteristics 8 and prevent stable production of cells for clinical use 9. The use of FBS also brings about the risk of contamination associated with harmful pathogens such as viruses, mycoplasma, prions, or unidentified zoonotic agents. Even if human serum is used, one cannot exclude the possibility of batch-to-batch variations and transmitted infections. Thus, the use of serum from humans and animals should be avoided.

Serum-free media are supplied by many manufacturers. For clinical use, confirming the lack of serum cross contamination is paramount for high quality control. Conventionally, Neu5Gc, a sialic acid derived from non-primates, is used as a marker for detecting bovine serum. However, Neu5Gc is not expressed on cells cultured using human serum 10. Thus, there is a need for new markers to help surmount these obstacles.

During studies on effect of Ad-MSC on pain using a human pain gene array containing 84 functional genes, we noticed that mammalian bradykinin receptor subtype B1 (BDKRB1) gene was constantly upregulated when serum was used in the culture medium. In this study, we attempted to establish further the potential of this gene as a new marker indicative of the presence of serum and facilitate a method for detection of human and animal serum in Ad-MSCs culture medium.

## Materials and methods

### Preparation of mesenchymal stem cells

Adult human adipose tissues were collected from volunteers undergoing orthopedic surgery following the ethical guidelines of the Sun Field Clinic in Tokyo, Japan. Informed consents were obtained from the volunteers before the surgical procedure. After surgical removal, adipose tissue was transferred into a sterilized 50 ml tube filled with lactate Ringer's solution at 4 ºC within 24 hours prior to use. The adipose tissue was minced into small pieces with sterilized scissors. Minced tissue was suspended in 0.1% collagenase Type I at 37ºC for 60 min with agitation to digest the extracellular matrix. Digested tissue was centrifuged at 1,000g for 5 min and supernatant containing lipid was discarded. Pellet containing cells and debris was suspended in 20 ml of phosphate buffered saline (PBS) and filtered with cell strainer to remove debris. The cell pellet was washed twice with PBS, before suspending in sf-DOT medium (BioMimetics Sympathies, Tokyo, Japan), plated into culture flask and maintained at 37ºC with 5% CO_2_. The medium was replaced initially after 48 hours, and every 3 days thereafter. Once the adherent cells were confluent, the cells were detached with TrypLE Express (Thermo-Fischer *Scientific*, Waltham, MA) and re-plated. Passage 3 or 4 cells were used, unless otherwise described.

### Extraction of mRNA

Cells were extracted with TRIzol (Thermo-Fischer Scientific) reagent. After chloroform addition to TRIzol, RNA was purified with ReliaPrep RNA Miniprep System (Promega Corp., Madison, WI) from aqueous phase according to the manufacture's instruction.

### Real-Time Quantitative RT-PCR (qRT-PCR)

The mRNA levels were quantified by real-time (RT) reverse transcription polymerase chain reaction (PCR) array on the RT^2^ Profiler PCR Array of Human Pain: Neuropathic & Inflammatory (SABiosciences, Frederick, MD) according to the manufacturer's instructions. This gene profiler array contained 84 functional genes comprising three categories of 1) conduction of pain: ion channels (TRPA1, TRPV1, TRPV3), sodium channels (SCN10A, SCN11A, SCN3A, SCN9A, SLC6A2), potassium channels (KCNIP3, KCNJ6, KCNQ2, KCNQ3), purinergic (P2Y) Receptors (ADORA1, P2RX3, P2RX4, P2RX7, P2RY1), opioid receptors (OPRD1, OPRK1, OPRM1), cannabinoid receptors (CNR1, CNR2); 2) synaptic transmission: Glutamate receptors (GRIN1, GRIN2B, GRM1, GRM5), serotonin (5-hydroxytryptamine) receptors (HTR1A, HTR2A), calcium channels (CACNA1B); 3) pain response modulation: eicosanoid metabolism (PLA2G1B, PTGER1, PTGER3, PTGER4, PTGES, PTGES2, PTGES3, PTGS1 (COX1), PTGS2 (COX2)), inflammation (ACE, ALOX5, BDKRB1, CALCA, CCK, CCKBR, CCL2 (MCP-1), CCR2, CD200, CD4, CHRNA4, CSF1 (MCSF), CX3CR1, DBH, EDN1, EDNRA, FAAH, GCH1, IL10, IL18, IL1A, IL1B, IL2, IL6, ITGAM, ITGB2, MAPK1 (ERK2), MAPK14 (p38ALPHA), MAPK3 (ERK1), MAPK8 (JNK1), PENK, PNOC, PROK2, TAC1, TACR1, TLR2, TLR4, TNF), neurotransmitters (ADRB2, COMT, DBH, MAOB, PDYN, PENK, PNOC), neurotrophins (BDNF, GDNF, NGF, NTRK1). Briefly, total RNA (1 μg) was reverse-transcribed into first-strand cDNA and used as a template to perform real-time PCR on the Agilent Mx3000p QPCR system (Agilent Technologies, La Jolla, CA). The data were analyzed using the comparative ΔΔCt method, according to the PCR Array Data Analysis on the SABiosciences website.

### PCR analyses

The cDNA was synthesized with random hexamer primer using PrimeScript (TaKaRa Bio, Japan). PCR amplification of BDKRB1 was performed using the primers 5'-gccttcattttctgcctgag-3' and 5'-aggctgcagcgagctatg-3'. As a control, PCR amplification of GAPDH was performed using the primers 5'-agccacatcgctcagacac-3' and 5'-gcccaatacgaccaaatcc-3'.

## Results

### Genes specifically responsive to FBS

**Figure 1:**
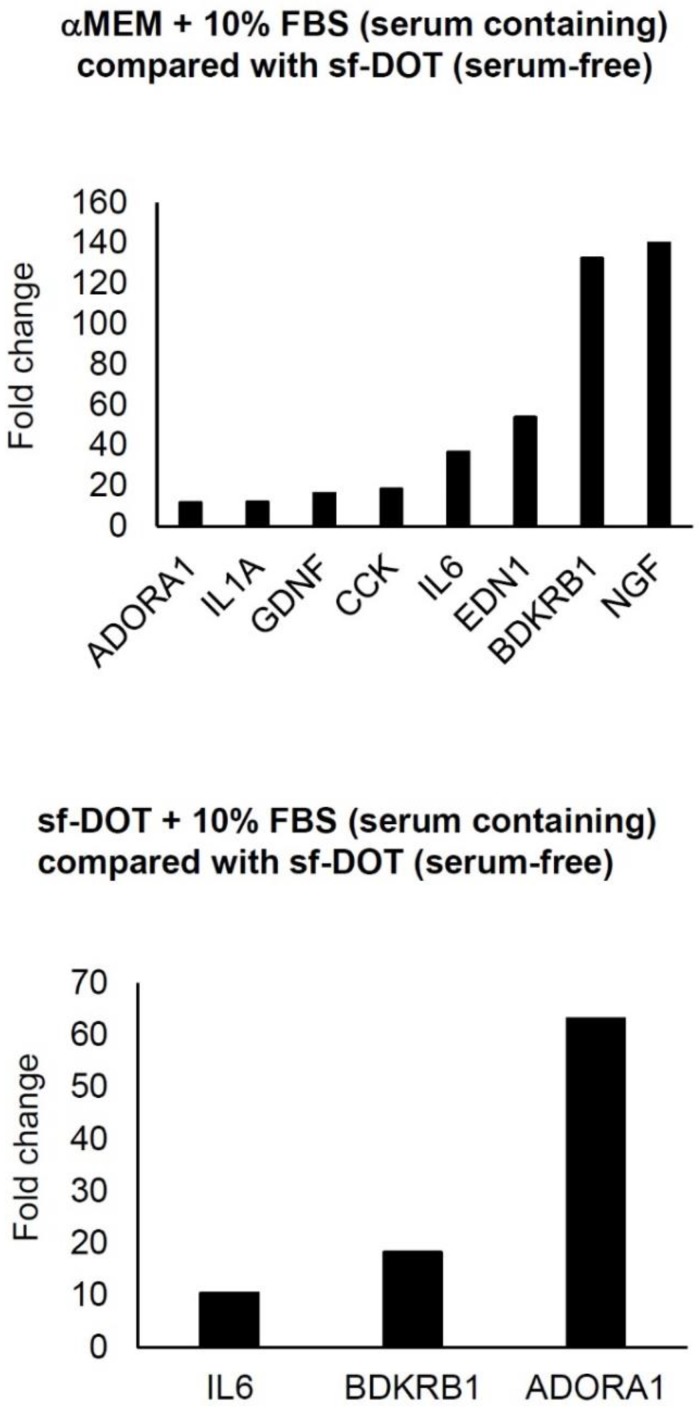
Result of qPCR Array. Expression of genes showing more than 10-fold increase. Upper panel shows comparison between human adipose-derived mesenchymal stem cells (hAd-MSCs) cultured with minimum essential medium α (αMEM) containing 10% FBS and sf-DOT (serum free). Lower panel demonstrates comparison between hAd-MSC cultured with sf-DOT containing 10% FBS and sf-DOT (serum free).

To identify gene(s) responsive to the presence FBS, qRT-PCR analysis using a human PCR array was performed. When Ad-MSCs were cultured in either sf-DOT or media supplemented with 10% FBS, the expression of three genes, IL6, ADORA1 and BDKRB1 were commonly upregulated compared with Ad-MSC cultured in sf-DOT without FBS **(Fig. 1)**. To confirm these results, PCR analysis was performed. Of the three genes, IL6 and ADORA1 genes were not detected by electrophoresis (data not shown), but BDKRB1 was confirmed as a specific serum induced gene.

### BDKRB1 in cells cultured in various serum-free media

**Figure 2:**
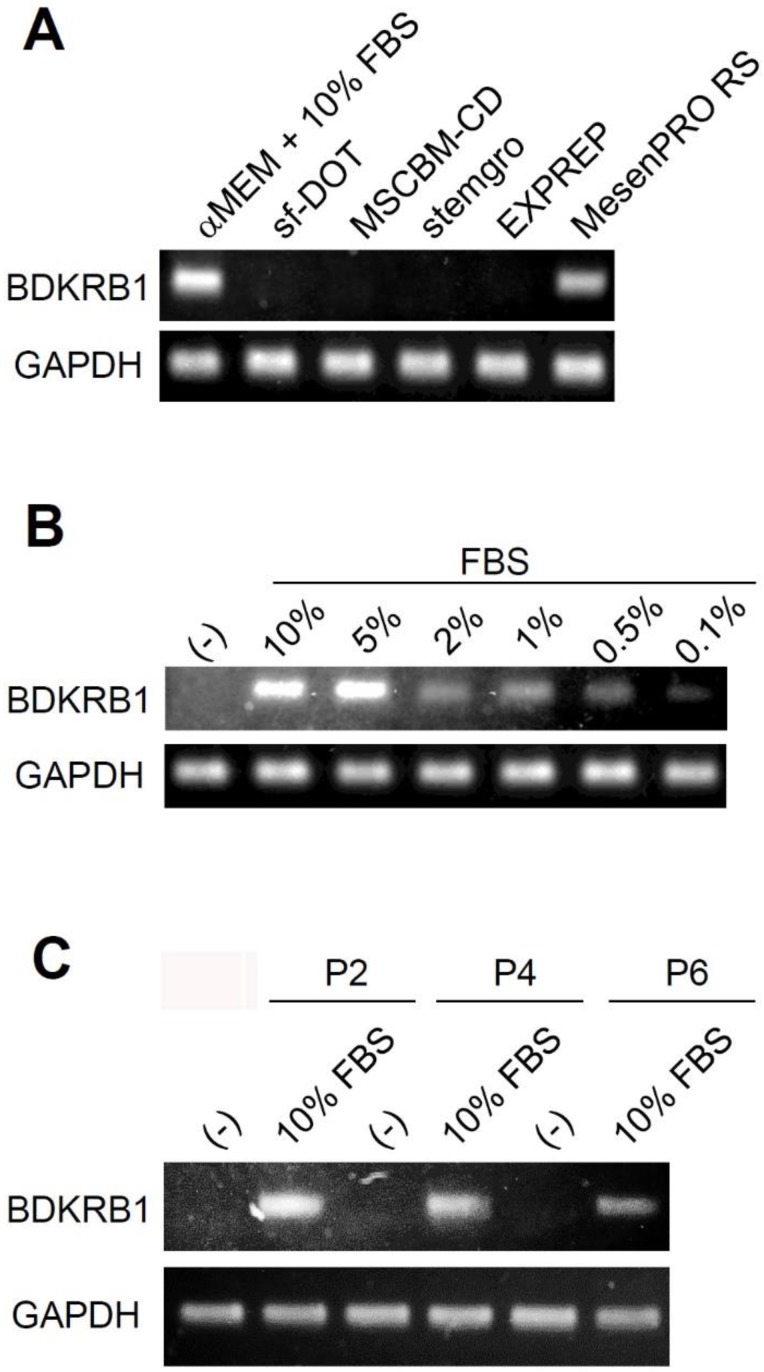
** A.** Expression of BDKRB1 in hAd-MSCs cultured with indicated media. **B.** Expression of BDKRB1 in Ad-MSCs exposed to various concentrations of FBS. **C.** Expression of BDKRB1 induced by FBS in multiple passages of hAd-MSCs.

Next, we examined whether the absence of BDKRB1 expression is a common feature when using other culture media. Ad-MSCs were cultured in several kinds of media available in the market such as minimum essential medium α (αMEM) containing 10% FBS, sf-DOT medium (BioMimetics Sympathies, Japan), MSCGM-CD (*Lonza*, Walksersville, MD), MesenGro (Corning, Tewksbury, MA), EXPREP (BioMimetics Sympathies, Japan), and MesenPRO RS (Thermo Fischer *Scientific)* for 3 days. Ad-MSC cultured in αMEM containing 10% FBS and MesenPRO RS expressed BDKRB1. The MesenPRO RS medium is a reduced serum medium containing 2% serum. These experiments confirmed that serum induces BDKRB1 expression in Ad-MSCs (Fig. 2A). To investigate the sensitivity of BDKRB1 expression in response to serum, the expression of BDKRB1 was measured in Ad-MSC cultured in serum-free medium and when supplemented with various concentrations of FBS. BDKRB1 expression was induced proportionally in response to serum concentrations and was detectable when cells cultured with 0.1% serum in the medium (Fig. 2B). The sensitivity of BDKRB1 in detecting serum was very high. Moreover, BDKRB1 expression induced by serum was stable across multiple passages of Ad-MSCs (Fig. 2C).

### BDKRB1 in the presence of human serum

**Figure 3:**
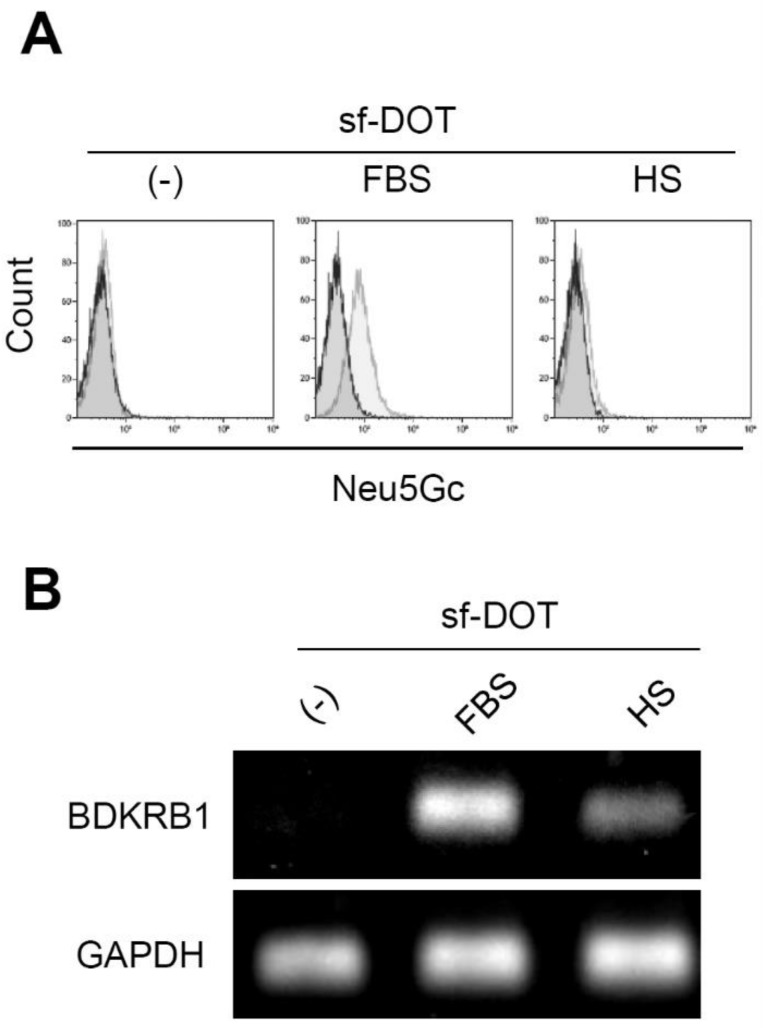
** A.** Surface expression of Neu5Gc on hAd-MSCs cultured in sf-DOT (left), when supplemented with 10% fetal bovine serum (FBS, middle) or with 10% human serum (HS, right). **B.** BDKRB1 expression in the same cells as in A.

Neu5Gc is a sialic acid contained only in non-human animals. Sugar chains containing Neu5Gc are present on the surface of cells cultured in the presence of serum derived from non-human animals. Thus, conventionally detection of Neu5Gc on the surface of cells has been used to determine the use of non-human serum, such as FBS. Anti-Neu5Gc antibody could react with Ad-MSCs cultured in FBS but not with Ad-MSCs cultured in human serum (Fig. 3A). However, in addition to animal serum, the expression of BDKRB1 was detectable in Ad-MSCs exposed to human serum (Fig. 3B). Thus, BDKRB1 expression serves as a marker that can detect both bovine and human serum.

### BDKRB1 in the presence of inflammatory signals

**Figure 4:**
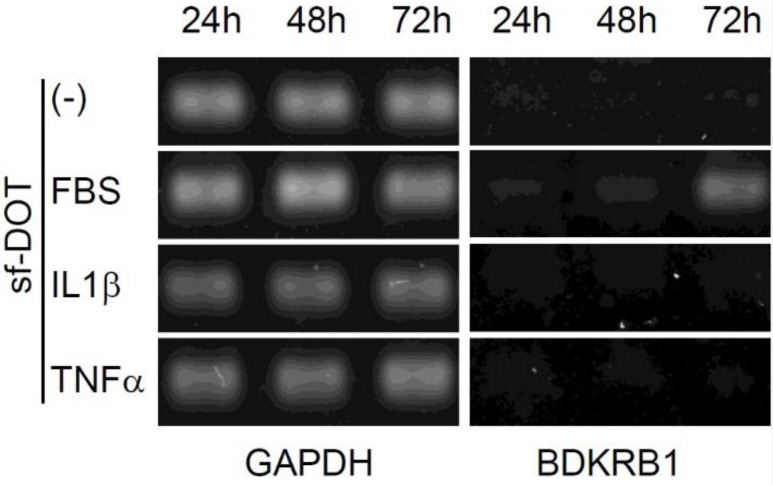
Expression of BDKRB1 in hAd-MSCs cultured with sf-DOT medium at 24, 48 and 72 hours when 10% FBS, 10 ng IL1β ml^-1^, or 10 ng TNFα ml^-1^ was added.

It has been reported that, BDKRB2 is constitutively expressed, whereas BDKRB1 is transiently induced by serum containing inflammatory signals, such as IL1β and TNFα at 24 hours 11, 12. Therefore, we examined the expression pattern of BDKRB1 in the presence of FBS, IL1β or TNFα. BDKRB1 expression was induced only by FBS and not by IL1β or TNFα (Fig. 4). BDKRB1 expression level was highest 72 hours after addition of FBS, suggesting that the mechanism of BDKRB1 upregulation by FBS is different from inflammatory signals.

## Discussion

Culture media for use with pluripotent stem cells have become an important issue. This is due to the establishment of embryonic stem (ES) cells 13 and induced pluripotent stem (iPS) cells 14 and high demands for use in basic and clinical regenerative medicine. In this regard, various kinds of serum-free media have been developed and are available in the market. Nevertheless, these media may contain undefined human or animal-derived components such as growth factors, hormones, various proteins and serum albumin (purified from blood) 15, 16. More specifically, undefined animal-derived constituents include lipids component of albumin which is considered as a contaminant 15, 16. On the other hand, a chemically defined media should contain all necessary components and their concentrations be identified and also proved to be free from human or animal-derived serum including albumin 15. Complete serum-free medium allows the establishment of a standardized protocol in compliance with good cell culture and quality control practices which is highly critical for cell-based therapy and regenerative medicine.

However, a method to determine if a cell culture medium contains serum has not been established yet and development of such method is of paramount importance. Conventionally, Neu5Gc, a sialic acid derived from non-human animals, is used as a marker to confirm if a medium contains bovine serum, however, Neu5Gc is not expressed on cells cultured using human serum 10.

In the present study, we identified that BDKRB1 gene is specifically induced by serum. Mammalian BDKRB1 and BDKRB2 receptors mediate the biologic functions of kinins. BDKRB1 is not detected on immune cells while BDKRB2 is universally expressed under physiological condition 11. In animal models of chronic inflammation, BDKRB1 is synthesized de novo following tissue injury and mediates hyperalgesia, whereas the BDKRB2 receptor appears to mediate acute inflammatory and algesic responses 12. As BDKRB1 is a receptor of bradykinin, it may be that the sensitivity to bradykinin is increased in the presence of serum.

In this study, we have not examined BDKRB1 expression in other culture cell lines including cancer cell lines. However, previous studies have detected BDKRB1 in breast cancer cell expression profiling 17, 18 and in hair follicles from males and females which are rich in stem cells 19, 20. In addition, our study was limited to only Ad-MSC cultures, nevertheless it can be highly expected that in other stem cell types, BDKRB1 would similarly respond to serum. Further studies are in progress to address the stem cell type generalization of our results.

In conclusion, our study facilitates a method for detection of serum in culture media. We established BDKRB1 as a specific genetic marker for the detection of human or animal serum in Ad-MSC culture media. Detection of BDKRB1 expression is a convenient method for attesting the lack of serum contamination in the medium with a high sensitivity. This method can be easily adapted for quality control of serum-free media.

## Acknowledge

The authors have a patent P2017-205071A pending. This research did not receive any specific grant from funding agencies in the public, commercial, or not-for-profit sectors.
